# Treatment efficacy and tolerability of intravesical Bacillus Calmette-Guerin (BCG) - RIVM strain: induction and maintenance protocol in high grade and recurrent low grade non-muscle invasive bladder cancer (NMIBC)

**DOI:** 10.1186/1471-2490-14-11

**Published:** 2014-01-27

**Authors:** Naim B Farah, Rami Ghanem, Mahmoud Amr

**Affiliations:** 1 From the department of surgery, section of Uro-oncology, King Hussein Cancer Center, Amman, Jordan; 2 Section of Uro-oncology, King Hussein Cancer Center, Amman, Jordan; 3 Department of Surgical oncology, Amman, Jordan

**Keywords:** Bladder cancer, BCG-RIVM, Intravesical, Maintenance

## Abstract

**Background:**

BCG-RIVM strain was used in many treatment protocols for non-muscle invasive bladder cancer only as induction courses. Cho et al. *(Anticancer Res 2012)* compared BCG-RIVM induction and 'standard' maintenance *(Lamm et al., J Urol. 2000)* to mitomycin C. They found no statistically significant differences regarding disease recurrence and progression. The purpose of our study was to determine the efficacy & tolerability of this specific BCG RIVM strain, using six-weekly, induction course and single monthly instillations as maintenance for one year, in high risk recurrent, multifocal low grade and multifocal high grade pTa/pT1, CIS transitional cell carcinoma of bladder.

**Methods:**

From 2003 - 2012, BCG-naive patients treated with intravesical BCG-RIVM for high-risk multifocal NMIBC were identified. Transurethral resection of bladder tumor (TURBT) and re-staging TURBT within six weeks, was done for accurate staging and complete elimination of disease. A six-weekly induction course, started 2-3 weeks after the last TURBT, followed by monthly maintenance protocol for one year. Recurrence, progression, cystectomy free survivals, cancer specific and over-all survival were determined.

**Results:**

Sixty evaluable patients - median age 63, median follow-up 3.98 years. Forty-two patients (70%) completed BCG-RIVM treatment as planned. BCG termination was necessary in 18 patients (30%). Recurrence occurred in 16 patients (26.7%) at a median follow-up of 24.2 months while progression occurred in five patients (8.3%) at a median follow-up of 33 months. Recurrence-free survival and progression-free survival rates were 73% and 92% respectively. Cystectomy was performed in seven patients (12%) with a cystectomy-free survival of 88%. There were no cancer specific deaths. Two patients died of other causes (3.3%). The overall survival rate was 97%.

**Conclusions:**

Our study is the first to show the clinical efficacy and tolerability of BCG-RIVM strain in the management of high risk NMIBC when given in a schedule of six-weekly induction with monthly maintenance for one year. Our maintenance protocol, achieved equivalent recurrence-free, progression-free, disease specific survival and overall survival to the reported literature and the more intense three-years South West Oncology Group (SWOG) protocol.

## Background

Bacille Calmette-Guerin for urothelial carcinoma has been the most successful use of immunotherapy to date and represents the standard of care for urothelial CIS and superficial bladder tumors [1]. Although BCG has been in use for over 35 years, the optimal dose and instillation schedule remains unclear. Nevertheless several meta-analyses showed the overall superiority of intravesical BCG with some form of maintenance therapy over chemotherapy in terms of recurrence-free and progression-free survival rates [2-5]. The two Dutch trials [6,7] that failed to show superiority of RIVM or Tice BCG over mitomycin C, could be explained by a sub-optimal BCG schedule, recruitment of high percentage of low risk patients, the type of BCG strain used or short follow-up. Other trials [8] that showed no advantage of maintenance BCG over induction course alone, may also be related to the BCG maintenance schedule, which may have t been sub-optimal for immunological boosting [9].

One of the major obstacles to a meaningful comparisons between different trials has been the adoption of variable maintenance schedules and BCG doses. Lamm et al. [10] advocated the use of an intense protocol of maintenance that he described as, "the current gold standard", which consisted of intravesical BCG - standard dose - every week for 3 weeks given at 3, 6, 12, 18, 24, 30 and 36 months from initiation of induction therapy; however, only 16% of patients completed the treatment protocol as planned.

When designing a maintenance protocol, two main factors should be considered: efficacy and compliance. Both of these factors depend on: BCG strain, dose volume, duration of treatment, total number and frequency of instillations [11].

Several different BCG strains are currently available for intravesical instillation [3,4,12]. These strains show differences in its phenotype, antigenicity and immune reactivity which may influence their antitumor activity, toxicity and clinical efficacy [13]. Despite BCG strain differences, very few studies were conducted to compare their efficacy, optimal dose and toxicity in the clinical setting.

Although BCG-RIVM is the third most commonly used BCG strain world-wide after Tice and Connaught [14], for unclear reasons, it is one of the less frequently used BCG strains in the reported clinical trials.

Since no clinical assumptions can be made regarding the individual BCG strain, unless efficacy and tolerability is proven in the clinical setting [5], we reviewed our experience in the use of this specific BCG -RIVM strain, with an induction course of one instillation every week for 6 weeks and a single monthly instillation as maintenance for one year, to determine its tolerability and efficacy in preventing recurrence, progression and cancer specific death and to determine the cystectomy-free survival and over-all survival.

## Methods

From January 2003 to December 2012 all patients who were treated with intravesical BCG-RIVM therapy at our institution were reviewed. All patients had histologically proven transitional cell carcinoma (TCC) of the urinary bladder pTa/pT1 with or without concomitant carcinoma in situ (CIS) or primary carcinoma in situ (2002 TNM classification of urinary bladder cancer and 2004 WHO grading system). Only two patients had Lymphovascular invasion. According to our institution's clinical practice guidelines, intravesical BCG-RIVM therapy was offered to all patients who were BCG naive, had high grade (TCC) Ta/T1 and CIS and to patients with low grade TCC but who were considered at high risk of recurrence and/or progression (multifocal and recurrent TCC). Complete transurethral resection of the bladder tumor (TURBT) and re-staging TURBT within six weeks prior to starting intravesical BCG therapy was done to all patients (including pT1 and pTa) to eliminate any residual tumor and confirm the stage of the disease. All patients had basic blood tests & computed tomography (CT) scan for chest, abdomen and pelvis and restaging CT scans & bone scan prior to radical cystectomy.

### Ethical approval

The study was approved by the King Hussein Cancer Center Institutional Review Board (No. 12 KHCC 70).

### Patients consent

Written informed consent to intravesical BCG therapy was obtained from all patients in this study. The consent was obligatory and a standard requirement in accordance with King Hussein Cancer Center Clinical Practice Guidelines.

### BCG protocol and treatment schedule

Intravesical BCG therapy was started 2 - 3 weeks after the restaging TURBT. The BCG strain used was: Seed RIVM (derived from seed 1173 -P2, 2 × 10 [8] -3 × 10 [9] viable units). Marketing authorization Holder and Manufacturer: Medac, Gesellschaft fur, klinischeSpezialpraparatembH, FehlandtstraBe 3, D-20354 Hamburg, Germany. BCG-RIVM strain was the only strain available at our institution. The choice was made on the basis of a competitive price, the commitment by the manufacturing company to ensure a steady supply of the medicine and on the presumption (at the time) that all commercially available BCG strains were equally effective.

The BCG-medac powder was re-suspended with 50 ml of 0.9% normal and introduced into the bladder via a 12 French urethral catheter.

Patients were instructed to hold on the medicine in the bladder for two hours and to lie on their back, right side, left side and face-down for 30 minutes each before voiding. They were also instructed to refrain from oral fluid intake four hours prior to and two hours after each treatment session. Induction course consisted of one instillations every week for 6 consecutive weeks, while maintenance course consisted of one instillations every month for one year in patients with no evidence of disease after the re-staging TURBT. Full BCG dose was used for both induction and maintenance therapy.

### Cystoscopy schedule

Cystoscopy +/- TURBT was done every three monthly for the first two years, then every six monthly for the next two years, then annually thereafter. If recurrence occurs then the clock for cystoscopy is re-set at every three months.

### Definitions of BCG responses

*BCG resistance:* defined as persistent disease at three months following induction course. *BCG refractory*: failure to achieve disease-free state at six months because of persistent or rapidly recurring disease. *BCG relapse*: recurrence of disease *after* six months of disease-free state*. BCG intolerance*: when patient discontinue treatment because of severe lower urinary tract side effects; (If this occurs during the induction course then it would not be considered BCG failure as treatment was not actually given). *BCG failure*: persistence of high grade disease at six months (or at three months if the initial tumor was pT1G3), any worsening of disease parameters (higher grade, stage, number of recurrences or appearance of CIS) or detection of pT2 disease during follow-up [15].

*Progression* was defined in this study as any worsening of disease parameters including change of pTa low grade to high grade or to pT1 or detection of pT2 disease during follow-up.

### Salvage therapy

Cystectomy was offered to patients who progressed, developed worsening disease parameters, were BCG resistant or BCG refractory. However for patients who declined cystectomy, intravesical therapy with MMC was offered. MMC treatment protocol was 40 mg in 40 ml of 0.9% normal saline one instillation every week for six weeks then one instillation every month for six months and a single instillation after every TURBT.

### Statistical method

Patients' characteristics such as age group, gender, histology and others were presented in terms of counts and percentages as shown in the tables and figures of this study. Treatment outcome (recurrence and progression) was compared among the treatment groups using Log rank test. Survival and event-free survival were presented by Kaplan-Meier curves showing their three/five year rates and standard error. Hazard ratios (HR) and their corresponding 95% confidence intervals (CI) were calculated to compare the risk between the different categories of each factor. All analyses were performed using: SAS version 9.1 (SAS Institute Inc, Cary, NC).

## Results

Sixty evaluable patients with a median age of 63 years (range 37 - 81; mean 61.7 years) were available. Median follow up was 3.98 years (range 8.28 - 93.6 months, mean 4.1 years). According to histological grade the study population of this cohort, consisted of two groups; **group 1:** 37 patients (61.7%) of **'high risk' low grade**, recurrent and multifocal TCC and **group 2:** 23 patients (38.3%) of **high grade** multifocal TCC. In 55 out of 60 patients (92%) the tumors were papillary, while primary CIS or concomitant CIS and papillary were present in 5 out of 60 (8%). A great emphasis in this study was placed on complete elimination of disease by re-staging TURBT prior to start of intravesical BCG therapy, close endoscopic surveillance and early counseling for radical cystectomy at the earliest sign of BCG failure.

Forty two out of 60 patients (70%) completed the intravesical BCG treatment protocol as planned, while BCG termination was necessary in 18 out of 60 patients (30%). The mean, median and minimum number of BCG instillations were 15.7, 18 and 3 respectively. In 16 patients, the terminations were related to sever lower urinary tract symptoms. BCG intolerance occurred in 11 of 18 patients (61.1%); BCG refractory state occurred in 3 of 18 patients (16.7%) and BCG resistance and sepsis in 2 of 18 patients each (11.1%). Complete demographic data are presented in (Table 1). The two patients who developed BCG sepsis, defined as: systemic BCG infection with persistent high grade fever and positive blood cultures for BCG; they required anti-tubercular therapy and further intravesical BCG was discontinued.

**Table 1:** Demographic data of study patients

				**N (Percent %)**
**Total number of patients = 60**				
Gender	Female			10(16.7%)
	Male			50(83.3%)
Smoking	Ex-smoker			33(55.0%)
	Non-smoker			9(15.0%)
	Smoker			18(30.0%)
No. of tumors	2-7			57(95.0%)
	>8			3(5.0%)
Stage	Primary CIS			2(3.3%)
	T1			24(40.0%)
	Ta			34(56.7%)
Grade	High grade			23(38.3%)
	Low grade			37(61.7%)
Histology	Papillary			55(91.7%)
	Concomitant CIS and papillary			3(5.0%)
	Primary CIS			2(3.3%)
BCG	BCG Complete			42(70%)
	BCG Termination			18(30%)
	BCG Termination reasons	BCG Refractory		3(16.7%)
		BCG intolerance		11(61.1%)
		BCG resistance		2(11.1%)
		BCG sepis		2(11.1%)
	Median	Maximum	Minimum	Mean
Age (Years)	63	81	37	61.7
Cigarette pack-year	60	150	10	55.4
No. of BCG installation	18	18	3	15.7
Follow up time (Years)	3.98	7.8	8.28 months	4.1

**Figure 1:**
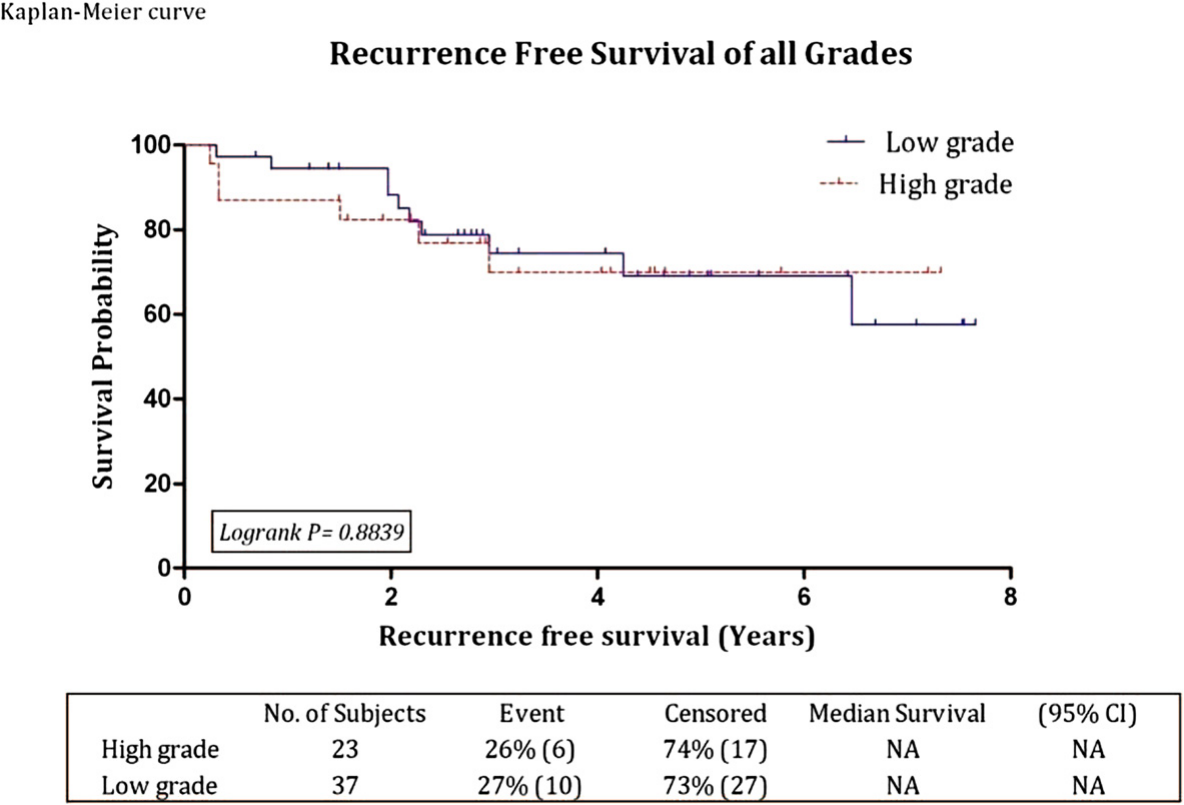
Kaplan-Meier recurrence free survival curve of all grades and 3 & 5 year survival rates.

**Figure 2:**
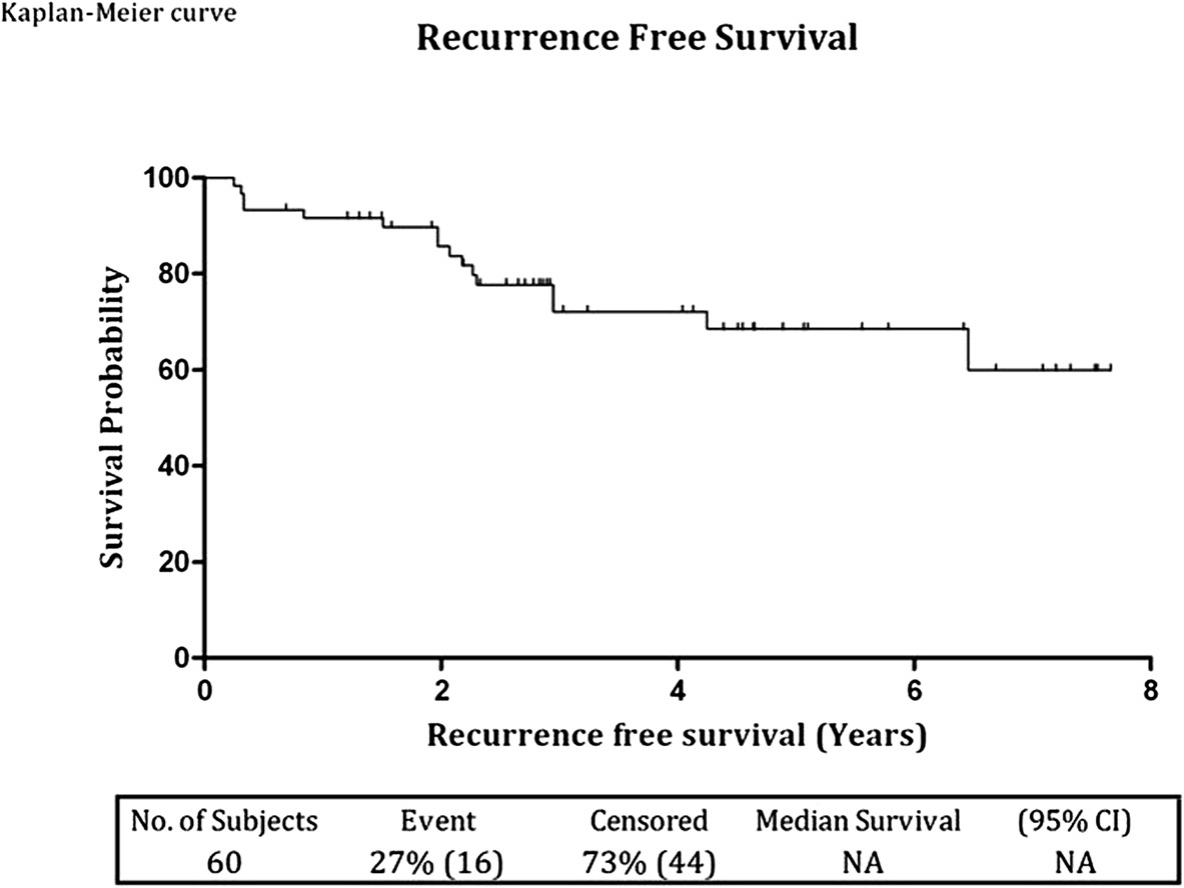
Kaplan-Meier recurrence free survival curve of all patients.

Forty four out of 60 patients (73.3%) remained free of recurrence during the entire follow up period while 16 out of 60 patients (26.7%) had recurrences at different time; five patients in the first year, 10 patients at 1 - 5 years and only one patient had a recurrence after five years. Median time to recurrence was 24.2 months (range 3.12 - 78). Recurrences occurred with equal frequency in the low grade and high grade groups, at 27% (10 of 37 patients) and 26% (6 of 23 patients) respectively (Figure 1). For low grade group, the recurrence-free survival rates at three and five years were 74% and 69% respectively, while the values for the high grade group were 69.9% for both periods. There was no statistical difference between the two groups (p = 0.88). When all grades where combined the three and five year recurrence-free survival rates were **72.5%** (95% CI: 57.67 - 82.98, SE: +/- 6.4) and **68.9%** (95% CI: 52.9 - 80.5, SE: +/- 7.05) respectively. Analysis of Kaplan-Meier curves for the recurrence-free survival of the whole cohort (Figure 2) and for the two study groups separately (Figure 1) showed that most recurrences (in 14 patients) occurred within the first three years, while recurrence occurred in one patient each at the 4th and 6th year.

**Figure 3:**
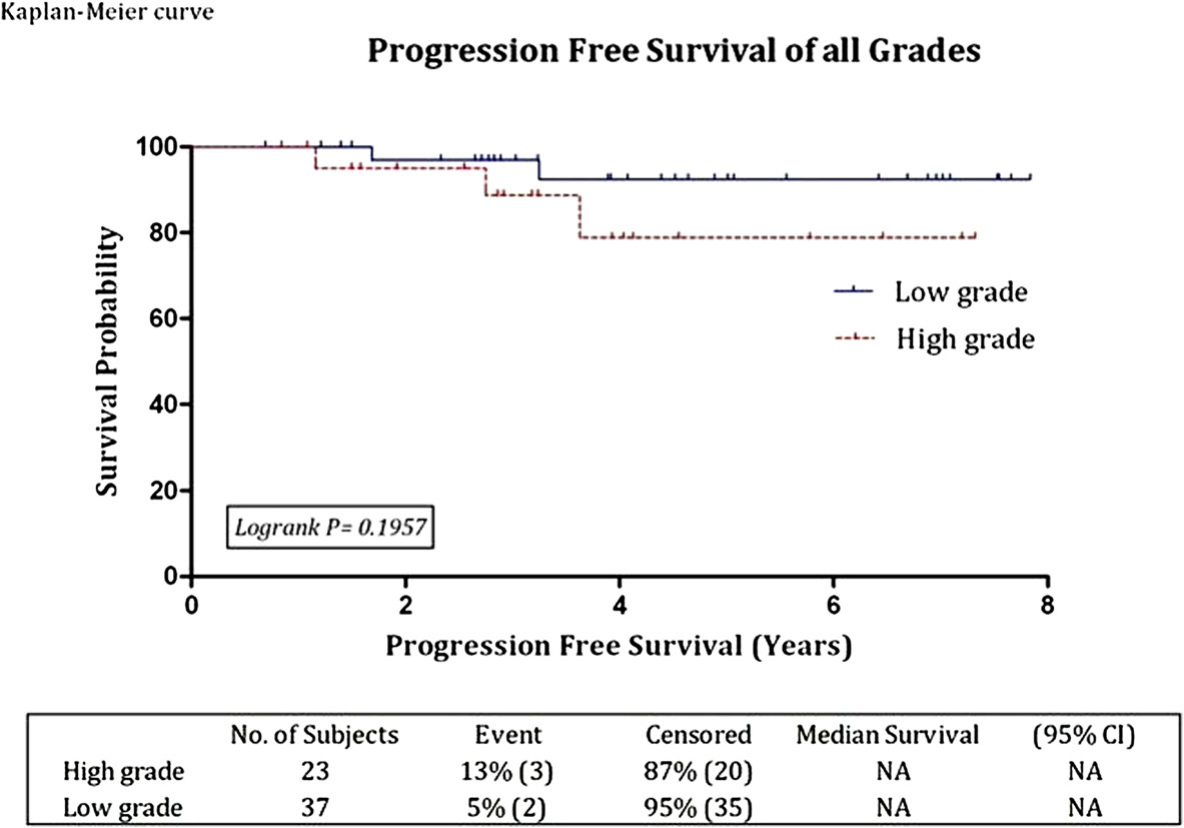
**Kaplan-Meier progression free survival curve of all grades.** Please note that low grade was also high risk.

**Figure 4:**
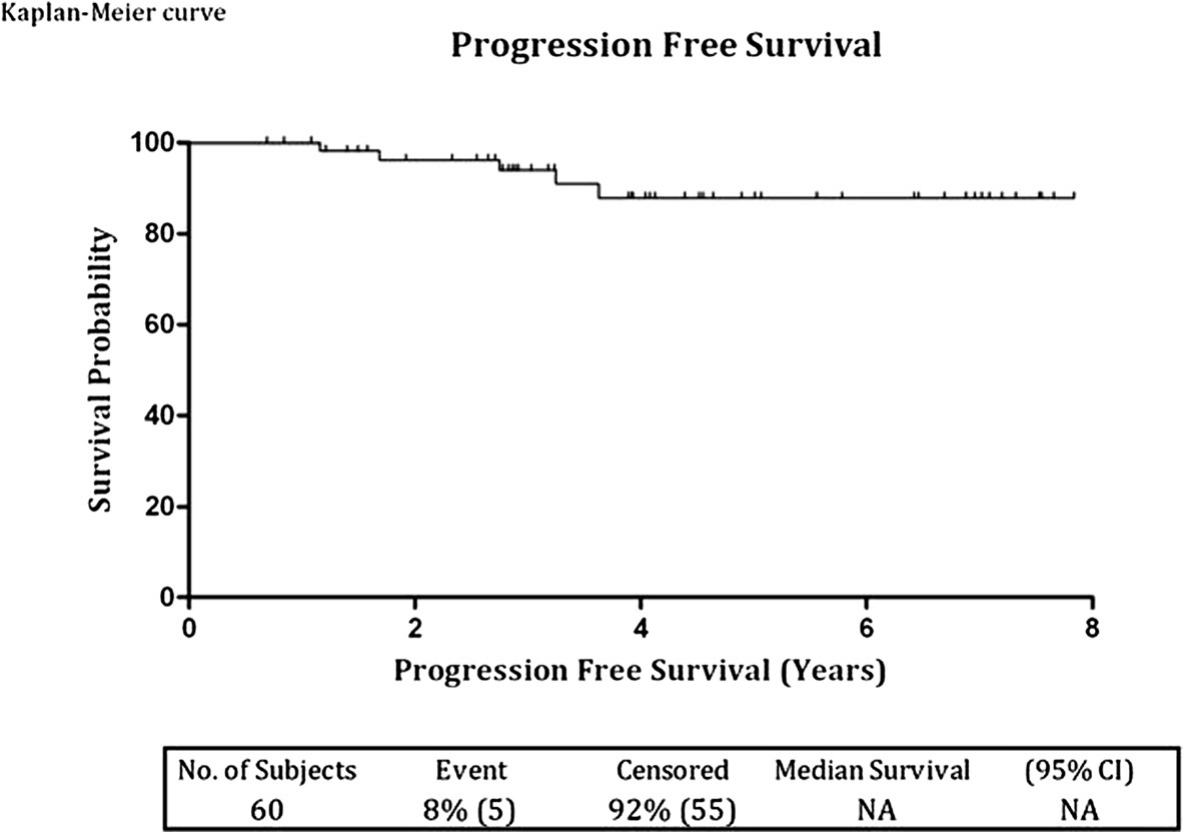
Kaplan-Meier progression free survival curve of all patients.

Progression occurred in five patients (8%) between one and four years after the start of BCG therapy. Median time to progression was 33 months (range 13.9 - 43.2). Three patients progressed from pTa/pT1 low grade to pT1G3 and only two patients progressed from T1G3 to pT2 disease; interestingly, both of these patients had Lymphovascular invasion at their initial histology. For the low grade group, the progression-free survival rates at three and five years were 96.9% and 92.4% respectively, while the values for the high grade group, were 88.7% and 78.8% respectively (Figure 3). There was no statistical difference between the two groups (P = 0.19). The three and five years progression-free survival rates for all grades combined were **93.9%** (95% CI: 82.4 - 98, SE: +/- 3.4) and **87.9%** (95% CI: 72.8 - 94.9, SE: +/- 5.2) respectively. Analysis of Kaplan-Meier curves for progression-free survival for both groups combined (Figure 4) and for the two groups separately (Figure 3) showed that all progressions occurred within the first 4 years. Although the five year progression-free survival rate of low grade and high grade groups were 92.4% and 78.8% respectively, with a hazard ratio of 3.07 (Figure 3), it did not reach statistical significance. However the number of progressions were too few for definitive conclusion - two out of 37 patients (5%) in the low grade group and three out of 23 patients (13%) in the high grade group.

**Figure 5:**
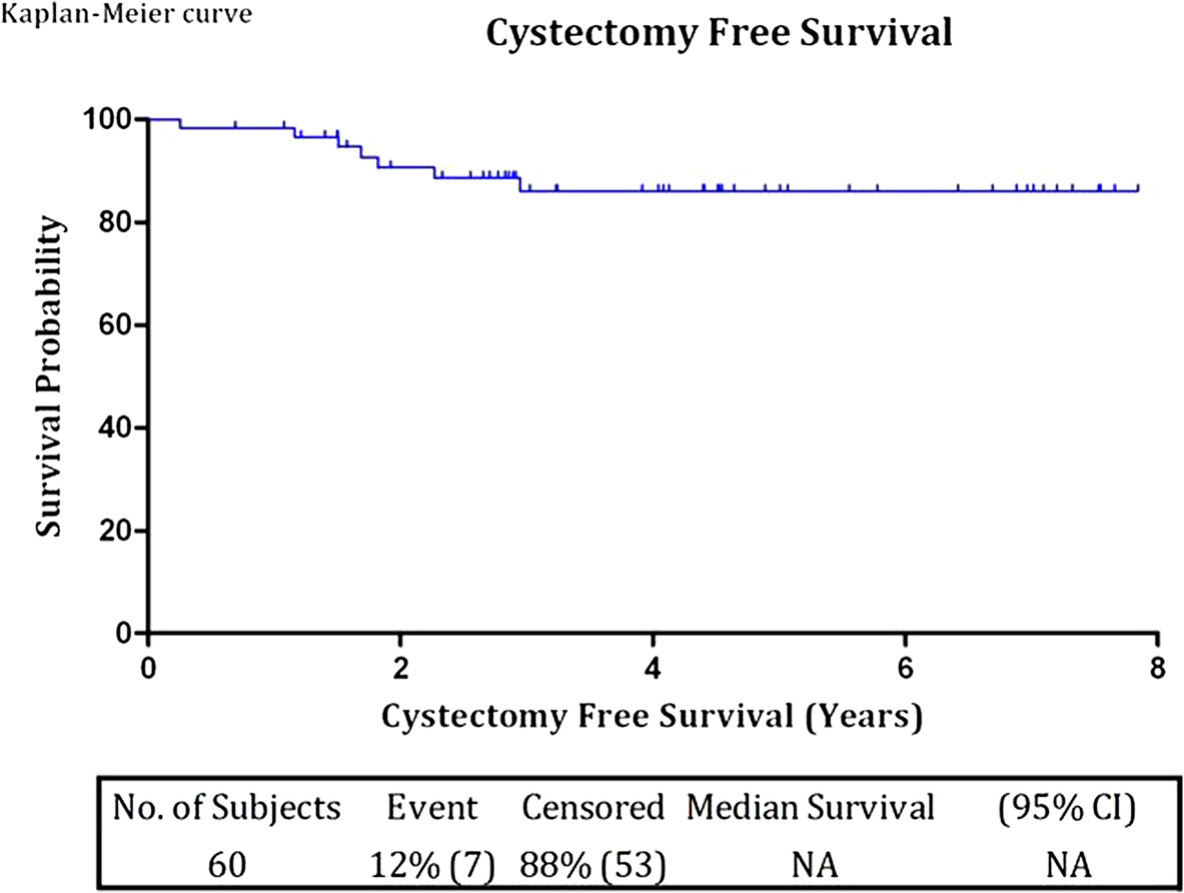
Kaplan-Meier cystectomy free survival curve during the entire follow-up period.

During follow up, patients who developed progression, BCG resistance, BCG refractory or BCG relapse were counseled and offered radical cystectomy. In this cohort radical cystectomy and bilateral pelvic lymphadenectomy - standard template - was performed in seven patients (12%), all within the first three years. The mean and median lymph node yield was 16 (range 8 - 29). Lymph nodes were negative in all patients. The final histology of the radical cystectomy specimens were as follows:- 2 patients: pT2 N0, 2 patients: CIS N0, 2 patients: pT0 N0 *(T1 G3 tumors were resected prior to the radical cystectomy)* and one patient: pTa G3 N0. The cystectomy-free survival rate was 88% (Figure 5). All cystectomy patients are currently alive; six patients without evidence of disease but one patient, during follow-up, developed retroperitoneal lymph node metastasis and was treated with systemic chemotherapy. One patient who progressed to pT2 disease (at 30 months of follow up) had radiologic evidence of pulmonary nodules, pelvic and retroperitoneal lymphadenopathy suggestive of metastasis; he was treated with systemic chemotherapy and is still alive at last follow-up with stable disease. Of the 12 patients who had recurrences but declined cystectomy at first sign of BCG resistance or refractory state, six patients were treated with intravesical MMC according to the above described protocol and six patients were kept on surveillance cystoscopy as they refused any form of intravesical therapy. Further recurrences occurred in eight of the 12 patients, of which one patient had radical cystectomy, two patients continued on MMC and five patients were kept on surveillance cystoscopy (Table 2).

**Table 2:** Summary of follow-up results

**Total number of patients**			**60**
Disease recurrence	No		44(73.3%)
	Yes		16(26.7%)
	Histology of recurrence	T1-High grade	4(25.0%)
		Ta-High grade	2(12.5%)
		Ta-Low grade	10(62.5%)
	Salvage therapy	Cystectomy	4(25.0%)
		Mitomycine C	6(37.5%)
		Surveillance cystoscopy & TURBT	6(37.5%)
Further recurrence	No		52(86.7%)
	Yes		8(13.3%)
	Histology further recurrence	T1-Low grade	1(12.5%)
		Ta-Low grade	7(87.5%)
	Further Salvage therapy	Cystectomy	1(12.5%)
		Mitomycine C	2(25%)
		Surveillance cystoscopy & TURBT	5(62.5%)
Disease progression	No		55(91.7%)
	Yes		5( 8.3%)
	Histology of progression	T1-High grade	3(60.0%)
		T2	2(40.0%)
	Further Salvage therapy	Cystectomy	2(40.0%)
		Surveillance cystoscopy & TURBT	2(40.0%)
		Systemic chemotherapy	1(20.0%)
Outcome	Alive		58(96.7%)
	Death from other cause ( one, myocardial infarction and one, sepsis secondary to perforated neobladder)		2(3.3%)

**Figure 6:**
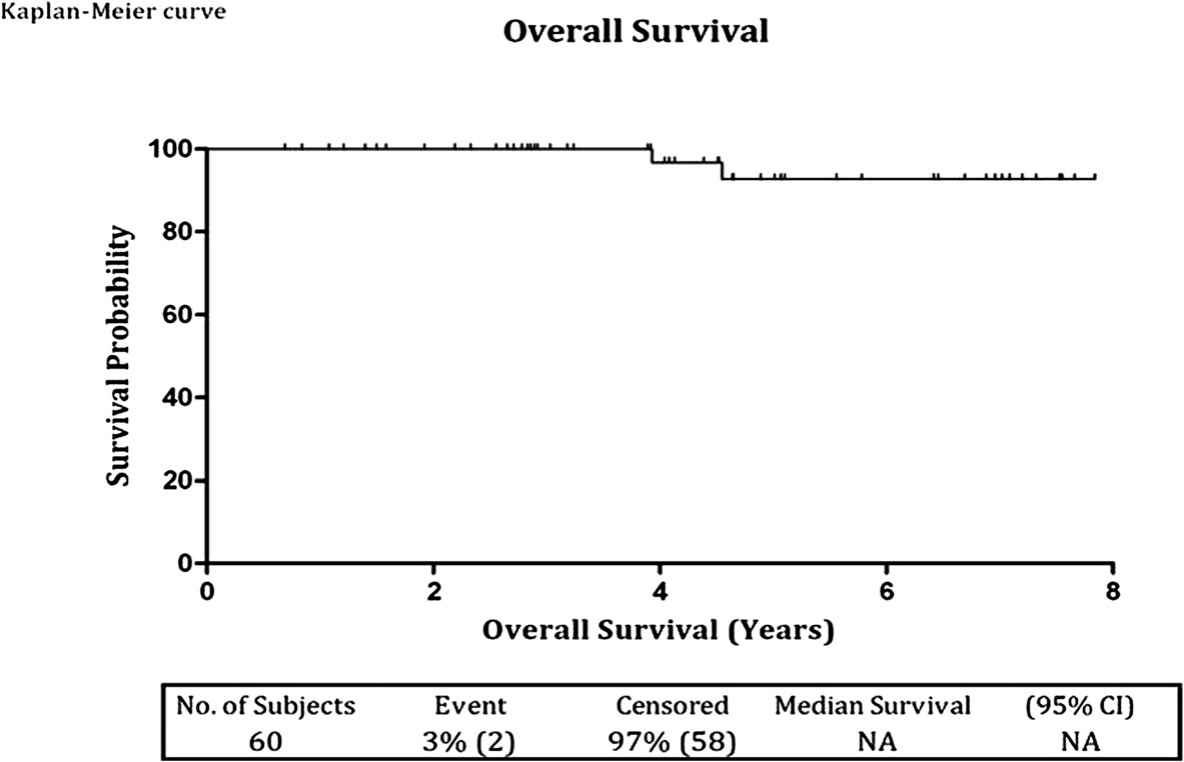
Kaplan-Meier overall survival curve.

There were no cancer specific deaths at a mean follow up of 4.1 years and the overall survival was 97% (Figure 6).

### Current status

At last follow up, 58 patients (96.7%) were alive. Two patients died (3.3%), one patient from myocardial infarction while the other patient died from bacterial sepsis secondary to perforated neobladder. The perforation occurred 11 months after the radical cystectomy with no evidence of recurrent disease; both of these deaths occurred around the 4th year of follow up. There were no cancer specific deaths in this cohort (Figure 1). The three and five year overall survival rates were estimated at **100%** (95% CI: NA and SE: NA) and **92.7%** (95% CI: 73.7 - 98.2, SE: +/- 4.98) respectively.

## Discussion

Several studies have established the superiority of intravesical BCG with maintenance over chemotherapy in intermediate and high-risk NMIBC with regard to long-term tumor recurrence, progression and mortality [9,10,16].

However, Malmstrom et al. [4] reported on an individual data meta-analysis of nine trials with 2820 patients, comparing long-term outcome of intravesical MMC versus BCG in NMIBC. All The trials included in that meta-analysis had some sort of maintenance MMC. The authors found that, for prophylaxis of recurrence, maintenance BCG was needed to be more effective than MMC. Prior intravesical chemotherapy was not a confounder, and did not bias the results in favor of BCG. However there were no statistically significant differences regarding progression, overall survival and cancer specific survival between the two treatments.

Although a majority consensus suggests that BCG with maintenance is superior to MMC in recurrence free survival, the debate is likely to continue, regarding progression and cancer specific survival, unless researchers address in their studies, the type of the BCG strain used among other variables.

The emphasis on the use of BCG with maintenance in the treatment of NMIBC was further highlighted by Saint et al. [2] who reported on a meta-analysis of 24 trials with some form of BCG maintenance involving 1685 patients. They found that, at a mean follow-up of 41 months the average recurrence free survival rate was 70%, the average progression rate was 8% and the disease-free specific survival rate was 91%. Our results are in concordance with the above meta-analyses and others (Table 3). The three and five year recurrence-free survival rates in our study were, 72.5% and 68.9% respectively, and the progression-free survival rates for the same periods were, 93.9% and 87.9% respectively. However, it is important to note that our cohort are *high grade and low grade with 'high risk'* for recurrence and progression because of the recurrent and multifocal nature of the disease (Table 1); thus accounting for a similar biological behavior in terms of recurrence and progression rates for the two treatment groups.

**Table 3:** Results of studies that used different BCG strains and maintenance protocols

**Author**	**No. of patients**	**Follow-up months (median)**	**Recurrence %**	**Progression %**	**Disease specific survival**	**BCG strain**	**Maintenance protocol**
Lamm 2000 [9]	192	98	40	24	*83*	Connaught	3 weekly at 3, 6, 12, 18, 24, 30, 36
Peyromaure 2003 [26]	57	53	42	23	88	Pasteur	3 weekly at 3, 6, 12, 18, 24, 30, 36
Pansadoro 2003 [27]	86	91	35	14	94	Pasteur	Every 2 weeks × 6, every month × 6, every 3 months × 6
Yoo 2012 [18]	92	43	35.6	20	85.3	Tice	Monthly for 12 months
Present series	60	47.8	26.7	8.3	100	RIVM	Monthly for 12 months

In our study only two patients progressed to muscle invasive disease during the follow-up period. One patient underwent immediate Radical cystectomy, while in the other patient, re-staging studies showed pulmonary nodules and retroperitoneal lymphadenopathy suggestive of metastatic disease and was treated with chemotherapy. Both of these patients had Lymphovascular invasion at the initial histology suggestive of a bad prognosis.

However, Cho et al. [17] reported in their series, the presence of Lymphovascular invasion in 29 out of the 107 patients (27%), but made no reference to this finding in the analysis of the results. In fact European Association of Urology Guidelines makes no reference to Lymphovascular invasion in the management of NMIBC.

Yoo et al. [18] reported on 92 patients treated with a monthly maintenance protocol (BCG-Tice strain) for one year, and showed similar median recurrence-free (RSF) and survival rates to that of the SWOG protocol (BCG-Connaught strain) [9]. In the SWOG study, the median RFS was 76.8 months with 83% 5-year survival rate, and for Yoo et al. [18] study the corresponding figures were 87 months and 85.3%. Our study also achieve similar results with a protocol of monthly maintenance (BCG-RIVM) for one year. In our series the overall survival was 97% with no cancer specific deaths and an even lower progression rates (8.2%), compared to SWOG [9]and Yoo series [18] which were (24%) and (20%) respectively. This difference could be related to the different BCG-RIVM strain in our study, racial (Jordanian population) or to our protocol which included a mandatory re-staging TURBT prior to BCG therapy.

Cho et al. [17] compared the efficacy of BCG-RIVM and MMC in 107 Korean patients with pT1G3 bladder cancer. The BCG arm consisted of 53 patients, of which 26 were treated with only 6-weekly induction course, and 27 patients treated with similar induction course plus three once-weekly maintenance at 3, 6, 12 and 18 months; while The MMC arm consisted of 54 patients, of which 29 were treated only with (30 mg) weekly instillations for 6 weeks, and 25 patients with similar 6-weekly instillations plus monthly instillation for one year. The authors then combined the data of both groups in each treatment arm for analysis. They found that the 2-year recurrence-free rate for BCG and MMC to be 45.7% and 52.1% respectively, while progression occurred in 9.4% and 7.4% of patients respectively. The authors concluded that there was no statistically significant difference between the two treatment arms in terms of recurrence and progression rates. The authors did not report the compliance rate in the BCG maintenance group. In our view, the results should be interpreted with caution, since half of the patients in each of the treatment arms were treated with induction courses only, which is now considered sub-optimal therapy [19] and would have undoubtedly, diluted the results. A more meaningful analysis would have been, a comparison between the two maintenance groups.

The fact that, so many treatment protocols existed in just as many trials confirms that the ideal protocol is still unknown. The mechanism of action of intravesical BCG is incompletely characterized. However BCG-induced anti-tumor activity, depends on both, local and systemic immunological responses [20]. After initiating intravesical BCG instillation, immune stimulation generally peaks at 6 weeks. However with subsequent instillations (as in maintenance therapy) the immune stimulation peaks at 3 weeks, and is suppressed at weeks 4, 5, and 6 [9,11]. This would explain the negative results of maintenance protocols that used repeated six-weekly instillations. However, whether the ideal 'immune stimulation schedule' is, every month, every 3 months or otherwise, remains to be determined for the individual patient and for the specific BCG strain in the clinical setting. Thus, the 'gold standard' for BCG maintenance schedule is yet to be determined.

BCG intolerance and the development of complications are important factors in further determining the ideal maintenance protocol. BCG tolerance appears to be relate to dose, BCG strain, frequency and total number of instillations. Badalament et al. [21] reported a 36% compliance - Pasteur strain - on a schedule of one instillation every month for 24 months, and in Palou et al. series [22] this value was 33.8% (Connaught strain) on a schedule of 6 instillation every 6 months for 2 years. However, in Akaza et al. series [8] the compliance was 65.5% using 1/2 dose of BCG Tokio strain at one instillation every month for 1 year.

In our study, 70% of patients completed the 'once monthly maintenance' protocol while in Yoo et al. [18] study (BCG Tice strain) using a similar protocol, 100% of patients completed the treatment as planned. Cho et al. [17] al also reported a 100% patient's compliance using BCG-RIVM, on a maintenance protocol based on SWOG recommendation but for only 18 months. Clearly, such variable results, reported from different geographical locations may also have a racial explanation among others.

To improve BCG tolerance without compromising efficacy, not only frequency and number of instillations, but also dose reduction was considered. Saint et al. [2] analyzed the impact of reduced BCG dose (1/3 - 1/2 dose) in 10 trials, seven of which had some form of maintenance therapy, and found that, at a mean follow-up of 40 months, the average recurrence-free survival rate was 62%, the average progression rate was 11% and disease-free specific survival rate was 90%. The authors concluded that low dose and full schedules, when maintenance therapy is used, gave similar results for recurrence and progression. The meta-analysis further confirmed that maintenance therapy was better than first induction courses as regards recurrences and better than first and second induction courses as regards progression. On the other hand, low dose schedule in first or second induction courses should be considered carefully as there were reports of 25% progression in high risk tumors, suggesting that high-risk tumors and CIS would not benefit from dose reduction [2].

Our study is the first in which this specific BCG RIVM strain was used in the form of induction with monthly maintenance protocol for one year in patients with high grade and high risk low grade NMIBC. The treatment protocol was well tolerated. However, two patients developed BCG sepsis requiring discontinuation of therapy and initiation of antituberculous treatment. The majority of complications which were encountered in our study were the less serious lower urinary tract symptoms. BCG-RIVM was reported to have the least number of complications compared with other BCG strain (A. Frappier, Tice, Connaught and Pasteur) (10). However, Strock et al. [23] reported a 1.8% (of 858 patients), unusual solitary BCG ulceration (10 mm), exclusively in males, and seen more frequently with BCG-RIVM than Tice. We have not encountered this complication.

In our study, close surveillance, re-staging TURBT and early counseling with view to radical cystectomy for BCG failures were important tools in preventing cancer specific deaths. This is evident by the finding of pT2 in only two of seven patients and negative lymph nodes on the final histology of all cystectomy patients. Some would argue that this is an over treatment, however, evidence suggests that patients who recurred within a year of initial BCG therapy did significantly worse, with disease-free rates of 34-43% at 24 months, indicating that additional immunotherapy may not be appropriate [24]. In a more recent study, a decrease in survival was reported in patients cystectomised for pT1 disease [25] suggesting that it could be related to the more common use of intravesical therapy, thus, delaying radical surgery.

In our study five of the seven cystectomies were done within the first two year (Figure 2). Radical cystectomy was done in 12% of our patients. Our result is in concordance with that published in the literature which ranged from 9% - 26% [10,26,27].

BCG-RIVM strain was introduced into clinical practice in 1986 after its proven safety and efficacy in an animal study [28]. Over the ensuing 10 years or so, several studies were reported most notably by the Dutch South-East Cooperative Urological group and to a lesser extent by the European Organization for Research and Treatment on Cancer- Genitourinary Group (EORTC), using BCG - RIVM strain as induction courses only. However, this specific BCG strain was less frequently used in subsequently published trials compared to other BCG strains

Our interest in this specific BCG-RIVM strain stems from the fact that it is the main BCG strain used in our country (Jordan) for intravesical therapy of NMIBC. Thus, determination of its efficacy and tolerability in the clinical setting with a schedule of 'induction with maintenance' was mandatory, since such data is lacking in the literature.

Clinical evidence suggests that certain BCG strains are more effective than others [6]. Research on two bladder cancer cell lines using BCG strain S4-Jena and BCG-Tice to assess their efficacy on cancer cell lines proliferation and apoptosis showed that T24 cells were responders for S4-Jena and Tice BCG, while Cal29 cells were responders for S4-Jena only. The authors concluded that S4-Jena strain may represent an effective therapeutic agent for NMIBC [29].

A clinical study comparing BCG Tice and Connaught in Switzerland [16], showed more than two-fold more common recurrences in patients treated with BCG Tice than Connaught, prompting the authors to wonder about the worldwide clinical impact on patients, and economic burden on healthcare systems of different countries. They went on to assess the global distribution of different BCG strains used to treat bladder cancer. They obtained information from 55% (140/252) of all countries (72% of global population and 98% of industrialized countries) and showed that BCG Tice and BCG Connaught were the most commonly used BCG strains worldwide (each used in 54 countries), followed by BCG RIVM (in 28 countries), Pasteur (in 17 counties), Danish (in 6 countries), China and Tokyo (in one country each). The authors concluded that based on the observed difference in tumor recurrence for the widely use BCG Tice and Connaught in NMIBC, a large proportion of patients are at risk of an inferior treatment.

Clearly, no presumptions can be made regarding the clinical efficacy and safety of an individual BCG strain unless proven by an induction and maintenance protocol in the clinical setting.

## Conclusions

Our study is the first to show the clinical efficacy and tolerability of BCG-RIVM strain in the management of high risk NMIBC when given in a schedule of induction with monthly instillations as maintenance for one year. Our maintenance protocol, achieved equivalent recurrence-free, progression-free, disease specific survival and overall survival to the more intense three-years SWOG protocol.

Only head to head comparative trials between different BCG strains in the treatment of patients with high risk NMIBC can determine their relative clinical efficacy, toxicity, appropriate dose, and ideal maintenance protocol for the individual BCG strain.

## Abbreviations

BCG: Bacillus Calmette-Guerin; CI: Confidence interval; CIS: Carcinoma-in-situ; MMC: Mitomycin C; RFS: Recurrence-free survival; SE: Standard error; SWOG: South West Oncology Group; TCC: Transitional cell carcinoma; TURBT: Transurethral resection of bladder tumor; NMIBC: Non-muscle invasive bladder cancer.

## Competing interests

The authors declare that they have no competing interests.

## Authors’ contributions

NBF: Enrollment of patients, carried out endoscopic procedures and other surgical operations, follow-up of patients and drafted the manuscript. RG: Carried out intravesical BCG instillations, participated in endoscopy procedures and other surgical operations, follow-up of patients, collection of data and participated in the manuscript drafting. MA: Participated in intravesical BCG instillations, collection of data, follow-up of patients and participated in the discussion of the manuscript draft. All authors read and approved the final manuscript.

## Pre-publication history

The pre-publication history for this paper can be accessed here:

http://www.biomedcentral.com/1471-2490/14/11/prepub
